# Engaging health sciences librarians on data ethics: case study on a pilot curriculum

**DOI:** 10.5195/jmla.2022.1418

**Published:** 2022-10-01

**Authors:** Nicole Contaxis, Fred WZ LaPolla, Genevieve Milliken

**Affiliations:** 1 Nicole.Contaxis@nyulangone.org, Data Librarian, Lead of Data Discovery, NYU Health Sciences Library, NYU Langone Health, New York City, New York.; 2 fred.lapolla@nyulangone.org, Research and Data Librarian, Lead of Data Education and Course Director, NYU Health Sciences Library, NYU Langone Health, New York City, New York.; 3 Genevieve.Milliken@nyulangone.org, Data Services Librarian, NYU Health Sciences Library, NYU Langone Health, New York City, New York.

**Keywords:** Data ethics, library school, professional development, education

## Abstract

**Background:**

Ethical decision-making regarding data collection, visualization and communication is of growing importance to librarians. Data ethics training opportunities for librarians, however, are uncommon. To fill this gap, librarians at an academic medical center developed a pilot data ethics curriculum for librarians across the US and Canada.

**Case Presentation:**

Three data librarians in a health sciences library developed a pilot curriculum to address perceived gaps in librarian training for data ethics. One of the team members had additional academic training in bioethics, which helped to provide an intellectual foundation for this project. The three-module class provided students with an overview of ethical frameworks, skills to apply those frameworks to data issues, and an exploration of data ethics challenges in libraries. Participants from library schools and professional organizations were invited to apply. Twenty-four participants attended the Zoom-based classes and shared feedback through surveys taken after each session and in a focus group after the course's conclusion.

**Discussion:**

Responses to the focus group and surveys indicated a high level of student engagement and interest in data ethics. Students also expressed a desire for more time and ways to apply what was learned to their own work. Specifically, participants indicated an interest in dedicating time for networking with other members of their cohort, as well as more extensive discussion of class topics. Several students also suggested creating concrete outputs of their thoughts (e.g., a reflective paper or final project). Finally, student responses expressed a strong interest in mapping ethical frameworks directly to challenges and issues librarians face in the workplace.

## BACKGROUND

Currently, there are limited options for librarians interested in continuing education and training in topics related to data ethics and librarianship. Available classes and programs give pride of place to patron privacy issues, which are important but relatively narrow in terms of larger ethical considerations and frameworks relevant to librarians [1, 2]. These courses are complemented by research, professional guides, and work groups in data ethics and libraries that similarly focus on issues related to privacy for library patrons [3–6]. While patron privacy is an important issue, these materials do not address other issues in data ethics, such as equity and representation in library data.

While there are substantial resources for librarians interested in providing or building data services [7–9], only a few mention data ethics. Data ethics is an interdisciplinary field that investigates moral problems related to data and includes issues associated with data collection, (re)use, storage, sharing, and communication [10]. These issues have received growing attention, especially in the wake of controversies such as Cambridge-Analytica [11] and the Facebook emotional contagion study [12]. Critical data studies is a theoretical approach that situates data in social and cultural contexts, often with a focus on relationships of power and agency [13, 14]. It is related to data ethics, and the distinctions between the two fields are often blurred. Recent scholarship in critical data studies has paid attention to racism, sexism, and other forms of discrimination mediated and propagated by data, ranging from algorithmic policing to biased web search results [15–18]. In response to research in data ethics and critical data studies, fields such as data science, computer science, machine learning, and artificial intelligence (AI) have developed ethics education programs for those who work closely with data [19–21].

In order to address ethical issues, particularly those outside of privacy concerns, we developed a pilot curriculum address perceived gaps in librarian training for data ethics, centered around three topics: ethical frameworks, the ethics of data collection and communication with data, including statistical analysis and visualization.

## CASE PRESENTATION

### Program Development

In October 2020, two of the three members of the pilot team presented “The Charts Are Off: Approaches to Ethical Decision Making in Data Visualization” to the MLA Pacific Northwest Chapter [22]. This presentation focused on ethical frameworks and ethical issues connected to statistical analysis and visualization, including issues like visualizing uncertainty in data. The success of the talk and interest in the topic led to applying for a Network of the National Library of Medicine (NNLM) Data Award Grant to fund a pilot class for medical librarians and LIS students. We proposed a three-session class, each consisting of a librarian-led lecture and featuring a guest lecturer working in the field of data ethics. We budgeted for participant stipends of $100 gift cards to encourage evaluation survey response and attendance for all three sessions.

#### Curriculum

The curriculum, comprised of three 1.5-hour sessions, was planned with three course objectives in mind:

1. Students would be able to identify major ethical frameworks for decision making (e.g., consequentialism, deontology, etc.)
2. Students would be able to apply major ethical frameworks to issues related to data management, sharing, and visualization
3. Students would be able to explain ethical considerations for ongoing challenges faced in libraries, including those related to data collection and communication specifically

These three objectives were met through three separate class sessions: Overview of Data Ethics and Ethical Frameworks; Ethical Data Collection; Ethical Data Communication. The syllabus and other course materials are hosted on Zenodo [23].

The first class, Overview of Data Ethics and Ethical Frameworks, provided background on the field of data ethics. It covered relevant information on regulations (e.g., informed consent practices, HIPAA), ethical values (e.g., beneficence, non-maleficence, justice, and autonomy) and ethical frameworks, defined as the philosophical perspectives used to analyze ethical problems and find acceptable solutions to those problems [24]. One of the ethical frameworks we discussed was consequentialism, which posits that an action can be judged solely on the consequences of that action, regardless of social mores. For example, a consequentialist may excuse an act that is potentially viewed as distasteful in a given community if that act led to positive consequences [25]. Other frameworks discussed include deontology, virtue ethics, and social contract theory. This session was not meant to be prescriptive, but rather was meant to provide background on how ethical problems can be analyzed and how decisions can be made.

Our second class was Ethical Data Collection and class topics included methods for data collection in health settings, decision making with collected data (e.g., funding and health policy), and research data management. Building on the first class session, it also included concepts of health equity, health disparities, social justice and Community-Based Participatory Research (CBPR) [26]. Further, during class students were divided into breakout sessions to discuss four ethical topics: Indigenous data collection, COVID tracing apps, LGBTQ+ data collection, and wearable health devices.

Our third and final class was Ethical Data Communication, which focused primarily on ethical considerations concerning rhetoric and data. By data communication, we mean publications like data visualizations as well as presenting the results of statistical analyses. This discussion emphasized how decision-making around uncertainty in data can lead to misleading conclusions, particularly among individuals with lower levels of numeracy. The session also explored equitable representation within data-driven communications, for example how visualization like color-coded maps (such as political affiliation maps) may create impressions that erase individuals not in the majority. All three sessions are described in further detail in Table 1.

**Table 1:** 

Session Title	Topics Covered	Activity Included
Overview of Data Ethics and Ethical Frameworks	Ethical frameworks, including deontology, virtue ethics, consequentialism, and social contract theory Background information on key regulations, including HIPAA and the Common Rules Ethical values including autonomy, beneficence, nonmaleficence, and justice Guest Speaker Topic: Overview of fairness, accountability, and transparency in algorithmic development and research	Mentimeter quizzes to reinforce learning and foster discussion. Questions focused on ethical frameworks and values that can be used to analyze ethical issues.
Ethical Data Collection	Data collection methods Data integrity and data management Disparities in clinical trials Data collection and community Ethics in survey design Guest Speaker Topic: “Boundaries of Responsibility Data Collection”, with case studies in obstetrics and cardiovascular genomics	Breakout groups based on 4 different topics: indigenous data collection, COVID trading apps, LGBTQ+ health, and wearing health devices.
Ethical Data Communication	Interpretation of statistical concepts like p-values, “significance”, and effect sizes Communication of Uncertainty Empathy in visualization Perspectives of the speaker Guest Speaker Topic: Implicit and explicit argumentation, and mitigating harm caused to people using data visualizations, people being represented by data visualizations, and people impacted by data visualizations.	Mentimeter quiz to reinforce lessons.

To encourage classroom participation, sessions included interactive portions, including the use of Mentimeter polls [27] (a web application for making slides with dynamic polling and question and answer functionality) or Zoom breakout rooms to discuss the specific, predetermined topics mentioned above. Each session included suggested readings, as well as a 30-minute guest lecture from a scholar associated with data ethics. Students were given access to the course syllabus and class materials prior to class. The guest speakers were meant to provide a researcher's perspective and a deep dive on a topic of interest (e.g., algorithmic fairness, health data collection in obstetrics and genomics, data visualization in historical research). Guest speaker topics are included in Table 1, and slides are included in the class materials on Zenodo [23].

#### Marketing

To recruit participants for our pilot curriculum, we contacted MLA caucuses, professional email lists, and library and information science graduate programs and also promoted the pilot program on social media. The call specified that a $100 gift card would be provided to all participants who attend all three sessions as compensation for their time as participants in the pilot. The application process consisted of a short REDCap survey, which we scored using a rubric. To generate a cohort of participants that reflected a diversity of professional experiences and perspectives, the rubric accounted for the applicant's professional role (e.g., librarian in different types of libraries, students, archivists). We also asked for a statement of interest as part of the survey. The volume of applications greatly outstripped our expectations; we received 199 applications for 22 available spots.

The application rubric used a 6-point scale to assess evidence of participant interest. Twenty-four individuals received a perfect score of 6; because of the flexibility of the online format and additional funding availability, we were able to accept all 24. Applicants came from libraries and library schools across the United States and Canada, with 5 from the Southern United States, 4 from the West, 1 from Western Canada, 8 from the Northeast or mid-Atlantic, and 2 from the Midwest, with others not responding as to location. Over one-third (38%) self-identified as being a member of an under-represented group in librarianship. Half (50%) of the class were LIS graduate students and 42% were practicing librarians or archivists; the others were a library staff-member and an incarcerated population services librarian.

#### Evaluation

After each session, we conducted an evaluation survey via REDCap and, one week after the conclusion of the series, we also held a focus group.

#### Surveys

We developed a post-session survey that measured participants' responses to topics we wanted to measure [28]. The survey design was informed by our past experience conducting evaluations of data workshops for librarians and information professionals [29] but was not validated.

Response rates for the 3 post-session surveys were: 83% (n=20) for survey 1, 67% (n=16) for survey 2, and 46% (n=11) for survey 3. The same instrument was administered after each session. Our surveys used four-point Likert scale questions, asking users to indicate the following: how likely they were to use the material learned in class; how likely they were to recommend the class session to peers; how useful they found in-class activities; how useful they found the guest lectures; and how useful they found the course materials. Percentages can be seen in Table 2, and de-identified data and the survey instrument are hosted on Zenodo [28].

**Table 2:** Survey evaluation data from the three class sessions. Numbers refer to average score for each category, so a 4 would mean 100% responded with a 4. Specific ranges and meanings of each range are included in the header of each column.

Class Session	Average Score Will Use Material (4 = Definitely Will, 1 = Definitely Won't)	Average Would Recommend Class (4 = Highly Recommend, 1 = Do not Recommend)	Average Material Level Rating (1 = too low, 2 = just right, 3 = too advanced)	Average Rated Activities Useful (4 = Very Useful, 1 = Not at all useful)	Average Rated Guest Lecture Useful (4 = Very Useful, 1 = Not at all useful)
Overview of ethical frameworks (n=20)	3.4 (sd = 0.8)	3.5 (sd = 0.8)	2.0 (sd = 0.3)	3.7 (sd = 0.5)	3.6 (sd = 0.6)
Ethical Data Collection (n=16)	3.1 (sd = 0.6)	3.2 (sd = 0.7)	1.9 (sd = 0.4)	3.3 (sd = 0.7)	3.5 (sd = 0.8)
Ethical Data Visualization and Communication (n=11)	3.5 (sd = 0.7)	3.4 (sd = 0.7)	2.1 (sd = 0.3)	3.2 (sd = 0.8)	3.8 (sd = 0.4)

#### Focus Group

After all three sessions were completed, a focus group was convened. A group of 11 participants spoke with a member of our library's faculty who had not taught any of the sessions. An additional staff member, also not involved with the sessions, was present as a note-taker. The focus group was held on Zoom and recorded. To analyze the transcripts of the focus group, we used an applied thematic framework [30] to identify themes and generate codes. We approached our data from a Social Constructionist framing [31], and believe that a focus group was an effective means of drawing out the shared meaning of the class experience. We attempted to minimize bias by having a librarian who did not teach the courses facilitate the focus group. The instructors of the class analyzed and coded the focus group responses and, as a result, some bias may have been introduced. We attempted to minimize this risk by having all three instructors generate themes, agree to definitions in a code book, and then achieve consensus in coding through iterative reading of the transcript (See Zenodo for code book) [28].

### Participant Desire for More Interactive Opportunities and Relationship Building

Several themes arose during analysis of the focus group [See Table 3 for full list]. Seven participants cited the use of breakout rooms in the middle of class sessions as a favorite element. For example, one student noted:

**“I would also add that the breakout sessions were helpful … I really resonated with my group discussion, which was for the indigenous data sovereignty, and hearing the different perspectives from everyone in my group.”**

**Table 3:** Themes Identified, Number Coded, and Exemplary Quotes

Theme	Number of Comments	Exemplar Quotes
Time Constraints	11	“I think it just went by very quickly, which I understand was just a time limit thing, so it was, it just felt like afterwards, we didn't really have a chance to actually relay what we talked about…”
Breakout Groups	7	“…the breakout sessions were helpful, especially when, during the second week, when we were put in groups based on a different topic, and I really resonated with my group discussion.”
Additional Topics	7	“it would be great to take the topics that we had in the breakout groups and expand those into full lessons”
Relationship Building	6	“…to be comfortable answering, and maybe being wrong, or genuinely having that trust relationship where you can make a mistake, and not feel like you're going to be judged for it, but also to learn from the people around it… it's really relationship building.”
Professional Applicability	4	“Like, how can we apply this to our work… it was like an experience that… I was teaching a session with undergrads, and they were like, what's DuckDuckGo? … And I'm like, how do I say this without sounding like I'm into conspiracy theories?”
Assignment	4	“I love the activity idea. Like it did feel really, like we did the readings and we talked, but it might be nice to have something tangible come out of these things.”
Additional Communication Infrastructure	4	“If there was a place where we could just continue the conversation after the synchronous sessions, maybe a Slack channel or something like that… That might be a google place to crowdsource and share resources.”
Guest Speakers	3	“I would say the guest speakers were probably also my favorite part because they sort of gave like the practical side of the information.”
Data Science	3	“…I would say the sort of review of statistics, going back to the basics to even really high-level theory… the last class was one of the most relevant review and discussion for me…”
Worthwhile for Time	3	“…this really felt like a short amount of time to me, and I feel like I definitely got lots of new information, and also things to research further…”
Ethical Frameworks	2	“I love the overview of the ethical frameworks…”
Accessibility and Technology	2	“Another point I thought of, just in terms of accessibility, just kind of thinking about the equity lens… but if there's a way for close captioning to also be just integrated.”
Readings	2	I feel like the readings were also a good amount of readings; it wasn't too much”
Engagement Tools (Mentimeter)	1	“Mentimeter, yes. Yeah, I found that was really helpful, especially in terms of engagement. I felt like not only was it a good chance for me to reflect on how I was practicing these things, but I also found it really fascinating to see everyone else's answers”
Data Collection	1	“I would also add that any time the lecture in the breakout room applied to a specific information context or library context was most useful to me, especially when talking about developing data collection tools”

Related to enthusiasm for breakout rooms were comments on the desire to connect on a personal level with other individuals who are interested in data ethics. Six comments reflected an interest in relationship building, which we defined during coding as “Discussion of networking, relationship building and trust building among class attendees.” For example, one student expressed:

**“But it might have been nice … if we could have had like a smaller, kind of social breakout room, like at the start of things, so we kind of all meet each other, and … like a little opportunity to kind of chitchat, build rapport with the cohort.”**

### Time Constraints and Worthwhile Experience

The most cited issue in our focus group was a lack of sufficient time to fully explore materials and discuss topics. Eleven comments reflected concerns about time constraints, which we defined as “Issues related to shortness of sessions, breakout groups, guest lectures, and the curriculum itself in ways that felt limiting or constrained.” This was reflected in comments like:

**“I totally understand that it is a three-class course, and I think that had a lot to do with the time constraints, but I agree that I felt often rushed in terms of covering the amount of content and, especially in the breakout rooms, that I would have liked to I guess connect with other people interested in the same topics…”**

Even with time constraints, students found the class to be a worthwhile experience. We defined the “worthwhileness” of time as: “The time commitment equals or exceeds perceived value.” Examples include:

**“I would definitely say yes, because this really felt like a short amount of time to me, and I feel like I definitely got lots of new information, and also things to research further, so obviously like, all of the things talked about in the class cannot be fully explained, or like discussed in the three classes, but I felt like I got a lot of things either that will come up later in my career, or will be things that I am interested in researching further and gaining more interest in, that I just like a snippet of in the class.”**

### Readings, Assignments, and Infrastructure

To supplement in-class learning, students were given optional readings prior to class. Generally, students were receptive to these materials, with two individuals mentioning positive views on them. Additionally, a handful of students expressed interest in additional channels to continue the conversation outside of class, for example, Slack or some other communication tool to keep in touch and chat. Finally, we were surprised to hear from four students that they would have liked some sort of assignment or project as a means of making what they learned more tangible.

**“Like it did feel really, like we did the readings and we talked, but it might be nice to have something tangible come out of these things. Like, even if it were just like a little paragraph, like hey, this is how I'm going to apply this thing in my work, or something that you kind of, my brain definitely, like I retain things better if I kind of have to immediately apply something I read about, or something we talked about.”**

In sum, student evaluations held several key takeaways, including that participants wanted time to build relationships with others interested in data ethics, activities and assignments to apply lessons, and to have lessons related back to their work or future work as librarians. Additionally, participants enjoyed guest speakers and alternative perspectives.

## DISCUSSION

### Lessons Learned

Students who were able to participate expressed a strong interest in the topic, as well as a desire for more time and space around lessons to help process information. Because data ethics in libraries is a broad topic that touches on complex issues and ethical paradigms, students need more time to think about and understand lessons, as well as more time to build trust with one another. This need for trust is especially relevant in the context of discussion-driven classes, where many topics may be sensitive for participants. As such, despite the desire to maximize pedagogical content, it is especially important for educators involved in data ethics pedagogy to set aside time for students to get to know one another and to lessen didactic time in favor of providing space for lessons to be integrated and discussed.

Data ethics training in other domains (e.g., data science, engineering) has found similar levels of enthusiastic engagement from students [20,21,32]. For example, in a short course on data ethics for data science students, authors observed students organizing events to teach others about Open Science during and after their course of study, but they noted a need for long-term evaluation to see if students remained engaged with the ethical issues in their field [32]. Our pilot and investigation into data ethics in librarianship would benefit from a similar evaluation, particularly after the pilot is expanded.

Moving forward, we hope to provide this class to more people, while also increasing the amount of time for students to discuss and digest the information provided. We observed substantial interest in the program with 199 applicants, and 260 downloads of class resources from our Zenodo repository, as of March 21, 2022. This highlights demand for education in data ethics. Long-term evaluation of student interest and implementation of materials, as noted in similar case studies in other fields, will also need to be addressed.

## LIMITATIONS

This case study was a pilot project, and as such was of an exploratory nature. As a result, it remains unclear how effective the curriculum might be in other contexts. In addition, the study involved a relatively small group of participants, with specific backgrounds and lived histories, and results may have limited transferability to other contexts and future classes [33]. Furthermore, the competitive application process, although not originally intended as part of the pilot, could have skewed evaluation results as those who participated are non-representative and had demonstrated significant interest in and dedication to the topic. The application process meant that our pool of students was highly motivated and had a strong ability to articulate their interest in data ethics, raising the possibility of selection bias. Given that students inherently have a wide range of aptitudes, interests, and levels of receptivity to different topics, it is reasonable to expect that among students who may be less interested in writing a compelling application, there might be less engagement with the topic. For example, a recent talk on algorithmic ethics instruction highlighted sharply dividing levels of student receptivity [34]. Because of this potential for bias, evaluations may have been more positive than with a student body more representative of the broader librarian population. Additionally, the course instructors served as qualitative coders of the focus group transcripts, which may limit the dependability of the themes uncovered, as the filter of our own perspective influences our understanding of the codes [33]. Finally, our survey of participant views was not validated, and as such is not generalizable and may not be a reliable measure of future behavior.

## CONCLUSIONS & FUTURE PLANS

Implementing the pilot curriculum, as well as feedback gleaned from participant evaluations, has made it clear that library professionals have an interest in discussing and learning about data ethics issues. Based on the interest and positive feedback expressed by participants, we plan to expand this pilot. First, we will be adding another class session that addresses issues related to data and race, ethnicity, gender, and sexuality explicitly. While those topics were introduced in the three pilot classes, we want to address them in their own session and include a guest speaker who can address these issues through concrete examples. Second, we plan to give the class series to a larger audience through a partnership with the Network of the National Library of Medicine's National Center for Data Services (NCDS) in April 2022. The NCDS series of classes will focus more directly on issues related to medical libraries and we will expand the amount of time allotted for discussion, questions, and interactive activities to help foster and build community among health sciences librarians. Furthermore, the team plans to leverage the partnership with the NCDS so that we can access additional resources, such as learning management systems (e.g., Moodle, On-Demand courses), which will allow students to use and share materials, post opinions, reflections, and responses to articles, events, and/or work-related ethical issues, and network with each other.

## Data Availability

The de-identified data that support the findings of this study are openly available in Zenodo, doi: 10.5281/zenodo.4686020. Data which could not be thoroughly de-identified (e.g., focus group transcripts) are not included.

## SUPPLEMENTAL FILES

**NA**

## References

[1] Yoose B. Library Patron Privacy: Cycles and Strategies [Internet]. Webinar presented at; 2020 Feb 4. Available from: https://www.youtube.com/watch?v=LpEY521ueMs.

[2] Hinchliffe LJ, Jones KM. Prioritizing Privacy: Data Ethics Training for Library Professionals [Internet]. 2021. Available from: https://prioritizingprivacy.org/.

[3] Yoose B. Balancing Privacy and Strategic Planning Needs: A Case Study in De-Identification of Patron Data. J Intellect Freedom Priv. 2017 Jul 7;2(1):15–22.

[4] Prindle S, Loos A. Information Ethics and Academic Libraries: Data Privacy in the Era of Big Data. J Inf Ethics. 2017 Fall;26(2):22–33.

[5] Briney, Kristin, Swauger, Shea, Yoose, Becky, Ockerbloom, John, Harper, Charlie, Levernier, Jacob, et al. A Practical Guide to Performing a Library User Data Risk Assessment in Library-Built Systems. 2020 [cited 2022 Mar 22]; Available from: https://osf.io/v2c3m/.

[6] Kim K, Shorish Y, Swauger S, Nowviskie B, Bateyko D, Stuit M, et al. DLF Privacy and Ethics in Technology Working Group [Internet]. Digital Library Federation; 2020. Available from: https://osf.io/bdyvq/.

[7] Read KB, Koos J, Miller RS, Miller CF, Phillips GA, Scheinfeld L, et al. A model for initiating research data management services at academic libraries. J Med Libr Assoc [Internet]. 2019 Jul 1 [cited 2021 Sep 9];107(3). Available from: http://jmla.pitt.edu/ojs/jmla/article/view/545. 10.5195/jmla.2019.545 PMC6579580 31258450

[8] Federer L, editor. The Medical Library Association guide to data management for librarians. Lanham: Rowman & Littlefield; 2016. 230 p. (Medical Library Association books).

[9] Thomas A, Martin ER. Developing a Community of Practice: Building the Research Data Management Librarian Academy. Med Ref Serv Q. 2020 Oct 1;39(4):323–33. 33085951 10.1080/02763869.2020.1826185

[10] Floridi L, Taddeo M. What is data ethics? Philos Trans R Soc Math Phys Eng Sci. 2016 Dec 28;374(2083):20160360. 10.1098/rsta.2016.0360 PMC5124072 28336805

[11] Confessore N. Cambridge Analytica and Facebook: The Scandal and the Fallout So Far. The New York Times [Internet]. 2018 Apr 4 [cited 2021 Sep 10]; Available from: https://www.nytimes.com/2018/04/04/us/politics/cambridge-analytica-scandal-fallout.html.

[12] Kleinsman J, Buckley S. Facebook Study: A Little Bit Unethical But Worth It? J Bioethical Inq. 2015 Jun;12(2):179–82. 10.1007/s11673-015-9621-0 25740760

[13] Iliadis A, Russo F. Critical data studies: An introduction. Big Data Soc. 2016 Dec;3(2):205395171667423.

[14] Dalton C, Thatcher J. What Does A Critical Data Studies Look Like, And Why Do We Care? [Internet]. https://www.societyandspace.org/articles/what-does-a-critical-data-studies-look-like-and-why-do-we-care. [cited 2020 Sep 4]. Available from: https://www.societyandspace.org/articles/what-does-a-critical-data-studies-look-like-and-why-do-we-care.

[15] Gebru T. Race and Gender: Data Driven Claims About Race and Gender Perpetuate The Negative Biases of the Day. In: The Oxford Handbook of Ethics of AI. Oxford University Press; 2020.

[16] Benjamin R. Race after technology: abolitionist tools for the new Jim code. Medford, MA: Polity; 2019.

[17] Noble SU. Algorithms of oppression: how search engines reinforce racism. New York: New York University Press; 2018. 229 p. 10.1126/science.abm5861 34709921

[18] Eubanks V. Automating inequality: how high-tech tools profile, police, and punish the poor. First Picador edition. New York: Picador St. Martin's Press; 2019. 271 p.

[19] Saltz JS, Dewar NI, Heckman R. Key Concepts for a Data Science Ethics Curriculum. In: Proceedings of the 49th ACM Technical Symposium on Computer Science Education [Internet]. Baltimore Maryland USA: ACM; 2018 [cited 2021 Sep 10]. p. 952–7. Available from: https://dl.acm.org/doi/10.1145/3159450.3159483.

[20] Bender EM, Hovy D, Schofield A. Integrating Ethics into the NLP Curriculum. In: Proceedings of the 58th Annual Meeting of the Association for Computational Linguistics: Tutorial Abstracts [Internet]. Online: Association for Computational Linguistics; 2020 [cited 2021 Sep 10]. p. 6–9. Available from: https://www.aclweb.org/anthology/2020.acl-tutorials.

[21] Grosz BJ, Grant DG, Vredenburgh K, Behrends J, Hu L, Simmons A, et al. Embedded EthiCS: integrating ethics across CS education. Commun ACM. 2019 Jul 24;62(8):54–61.

[22] LaPolla FW, Contaxis N. The Charts are Off: Approaches to Ethical Decision-Making in Data Visualization [Internet]. Webinar presented at: NNLM Research Data Management Webinar Series; 2020 Oct 14 [cited 2021 Sep 10]. Available from: https://news.nnlm.gov/sea/2020/10/14/the-charts-are-off-approaches-to-ethical-decision-making-in-data-visualization/.

[23] Contaxis, Nicole, LaPolla, Fred, Milliken, Genevieve, Samuel, Kiran, Owens, Kellie, Church, Chris, et al. Ethical Considerations of Data: A Curriculum for Health Sciences Librarians (Pilot). 2021 Apr 7 [cited 2022 Mar 22]; Available from: https://zenodo.org/record/4686020.

[24] Bonde S, Firenze P. A Framework for Making Ethical Decisions [Internet]. Brown University: Science and Technology Studies. 2013 [cited 2022 Mar 16]. Available from: https://www.brown.edu/academics/science-and-technology-studies/framework-making-ethical-decisions.

[25] Sinnott-Armstrong W. Consequentialism. In: The Stanford Encyclopedia of Philosophy [Internet]. Fall 2021. Available from: https://plato.stanford.edu/cgibin/encyclopedia/archinfo.cgi?entry=consequentialism.

[26] Tremblay M-C, Martin DH, McComber AM, McGregor A, Macaulay AC. Understanding community-based participatory research through a social movement framework: a case study of the Kahnawake Schools Diabetes Prevention Project. BMC Public Health. 2018 Dec;18(1):487. 29650020 10.1186/s12889-018-5412-y PMC5897940

[27] Mentimeter [Internet]. Available from: https://www.mentimeter.com.

[28] Contaxis, Nicole, Milliken, Genevieve, LaPolla, Fred. Engaging Health Sciences Librarians on Data Ethics: Case Study on a Pilot Curriculum (Dataset) [Internet]. Zenodo; 2021 [cited 2022 Mar 22]. Available from: https://zenodo.org/record/5569742. 10.5195/jmla.2022.1418 PMC10124593 37101925

[29] Surkis, PhD, MLS A, LaPolla, MLS FWZ, Contaxis, MLIS N, Read, MLIS, MAS KB. Data Day to Day: building a community of expertise to address data skills gaps in an academic medical center. J Med Libr Assoc [Internet]. 2017 Apr 4 [cited 2021 Sep 10];105(2). Available from: http://jmla.pitt.edu/ojs/jmla/article/view/35. 10.5195/jmla.2017.35 PMC5370612 28377684

[30] Guest G, MacQueen K, Namey E. Applied Thematic Analysis [Internet]. 2455 Teller Road, Thousand Oaks California 91320 United States: SAGE Publications, Inc.; 2012 [cited 2021 Sep 10]. Available from: http://methods.sagepub.com/book/applied-thematic-analysis.

[31] Leavy P. The Oxford handbook of qualitative research. Oxford New York: Oxford University Press; 2014. (Oxford library of psychology).

[32] Bezuidenhout L, Quick R, Shanahan H. “Ethics When You Least Expect It”: A Modular Approach to Short Course Data Ethics Instruction. Sci Eng Ethics. 2020 Aug;26(4):2189–213. 32067185 10.1007/s11948-020-00197-2 PMC7417416

[33] Nowell LS, Norris JM, White DE, Moules NJ. Thematic Analysis: Striving to Meet the Trustworthiness Criteria. Int J Qual Methods. 2017 Dec;16(1):160940691773384.

[34] Ramachandran S, Cutchin SM, Fu S. Raising Algorithm Bias Awareness Among Computer Science Students Through Library and Computer Science Instruction. Virtual: July 2021. Available from: https://peer.asee.org/37634.

